# Right ventricular pressure overload directly affects left ventricular torsion mechanics in patients with precapillary pulmonary hypertension

**DOI:** 10.1371/journal.pone.0232544

**Published:** 2020-05-12

**Authors:** Ralf Kaiser, Dan Liu, Paula Arias-Loza, Kai Hu, Katharina Grotemeyer, Peter Nordbeck

**Affiliations:** 1 Department of Internal Medicine V, Saarland University Medical Center, Homburg / Saar, Germany; 2 Department of Internal Medicine I, University Hospital Würzburg and Comprehensive Heart Failure Center, Würzburg, Germany; 3 Department of Internal Medicine II, Saarland University Medical Center, Homburg / Saar, Germany; Scuola Superiore Sant'Anna, ITALY

## Abstract

This study examined the impact of septal flattening on left ventricular (LV) torsion in patients with precapillary pulmonary hypertension (PH). Fifty-two patients with proven precapillary PH and 13 healthy controls were included. Ventricular function was assessed including 4D-measurements, tissue velocity imaging, and speckle tracking analysis. Increased eccentricity index (1.39 vs. 1.08, p<0.001), systolic pulmonary artery pressure (64 vs. 29mmHg, p<0.001) and right ventricular Tei index (0.55 vs. 0.28, p = 0.007), and reduced tricuspid annular plane systolic excursion (19.0 vs. 26.5mm, p<0.001) were detected in PH patients as compared to controls. With increasing eccentricity of left ventricle, LV torsion was both decreased and delayed. Torsion rate paralleled this pattern of change during systole, but not during diastole. In conclusion, right ventricular pressure overload directly affects LV torsion mechanics. The echocardiographic methodology applied provides novel insights in the interrelation of right- and left ventricular function.

## Introduction

Left and right ventricle reside in the confined space of the pericardium. In response to right ventricle enlargement, the interventricular septum (IVS) will shift leftwards [1]. This phenomenon can often be seen in patients with chronic right ventricular pressure overload due to precapillary pulmonary hypertension (PH) and is recognized as D-sign in short axis view of echocardiography, referring to the flattened interventricular septum. It can be quantified by the ratio of the longer and shorter diameter of left ventricular (LV) chamber, known as eccentricity index (EI).

Previous echocardiographic studies in pulmonary hypertension mainly focused on right ventricular mechanics changes, relatively little is known regarding the interventricular interaction and the mechanical effects of the D-sign on the function of left ventricle. The D-sign not only reflects shear mechanical impact of the pressure overloaded right ventricle, but might also impair interventricular interaction. It might be speculated that compression of the left ventricle due to enlarged right ventricle might also diminish left ventricular function, the findings of preliminary earlier studies hinted this speculation [2]. However, comprehensive investigations facilitating the most recent echocardiographic techniques have not been reported yet.

Earlier studies focusing on the interrelation of left and right ventricular function revealed that about 20% to 40% of the right ventricular systolic pressure and volume outflow result from left ventricular contraction [2]. Functional investigations of the left ventricle however until then usually emphasized either on radial or longitudinal function. The Teichholz formula of ejection fraction is still one of the widely used screening parameters for left ventricular function. Its limitations result from the fact that only the narrowing of two points of the interventricular septum and left ventricular posterior wall are used in the calculation.

To overcome this limitation, planimetric methods such as the Simpson formula have been developed, taking into account the radial function as well as the longitudinal shortening of the left ventricle for better evaluation of left ventricular function. Newer techniques have been developed to recognize and better differentiate the individual disturbed components contributing to diminished ventricular function. Nowadays, the field of vectorized deformation techniques came into the focus of echocardiographic research. Spatial deformation of the myocardium can be divided into longitudinal, radial and circumferential components, and these components could be evaluated by novel techniques such as tissue velocity imaging (TVI) or speckle tracking echocardiography (STE). In short axis views of the left ventricle, STE can be used to quantify the rotation of the basal, middle and apical segments around the longitudinal axis through evaluating the contraction mode of the inner and outer myocardial layers. These layers contain diagonal fibers, and contraction of diagonal fibers could lead to both longitudinal shortening and twisting motion of the left ventricle. By measuring the apical and basal rotation in relation to the mid segments, torsion can be quantified as the difference of both movements in degrees. This motion contributes up to 20% of left ventricular systolic function and might be essential for evaluation of diastolic function. From this raw measurement, torsion is derived by normalization of ventricular length. The temporal derivations of these parameters are known as torsion rate and twist rate, respectively. Association of systolic torsion rate with left ventricular inotropy and maximal untwisting rate with left ventricular diastolic function have been described before [3, 4].

In the current study we hypothesized that the shift of the interventricular septum (flatten) not only alters morphology but also disturbs the functional integrity of the left ventricle. First, the natural moving mode of the diagonal fibers might be altered by local impression of the wall, resulting in passive muscular insufficiency of the fibers. Second, the rotational movement of each cross-section might be compromised due to aberration from the usually round geometry. Therefore, we examined the impact of septal flattening on alterations in left ventricular torsion in patients with precapillary PH. In clinical practice, assessment of left ventricular torsion might add to the outcome evaluation of precapillary PH patients.

## Materials and methods

### Participants

In this retrospective cohort study, 52 patients with proven precapillary PH were included during standard echocardiographic work-up. Patients were hemodynamically stable and compensated. PH diagnosis was based on previous diagnostics including right heart catheterization. The group comprised 22 patients with pulmonary arterial hypertension (PAH), 21 patients with chronic thromboembolic PH (CTEPH) and 7 patients with interstitial lung disease-associated PH (ILD). The diagnostic algorithm was consistent with current international guidelines. Patients with left-sided heart diseases were excluded. Thirteen healthy volunteers were included as control group.

### Data acquisition

**Fig 1:**
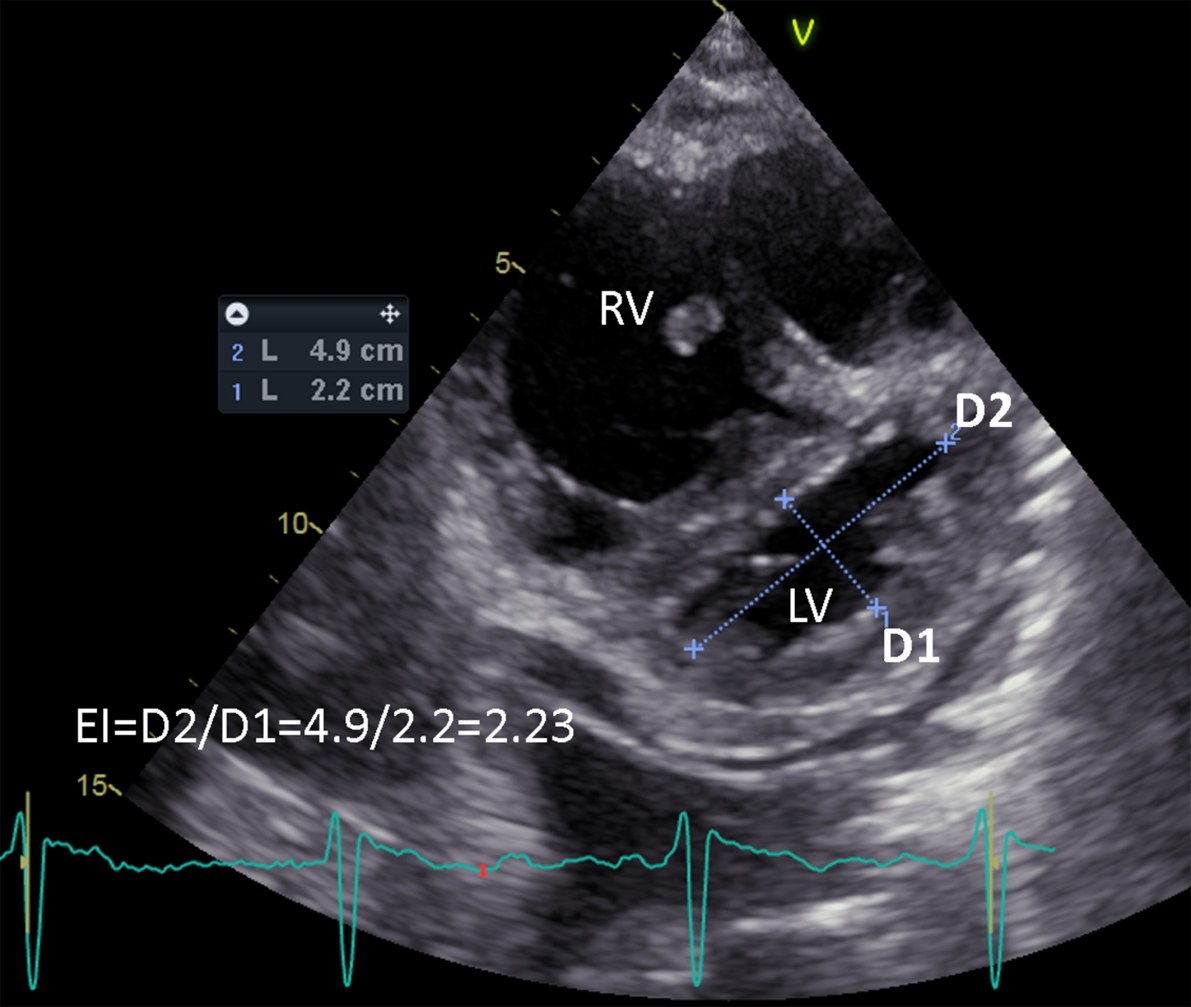
Measurement of echocardiography-derived Eccentricity Index (EI) at end-systole. EI is defined as the ratio of D2/D1, where D1 is the left ventricle short-axis diameter perpendicular to the septum and D2 is the left ventricle short-axis diameter parallel to the septum. LV, left ventricle; RV, right ventricle.

All participants received standard echocardiography. Standard parameters were acquired according to international guidelines as published by the American Society of Echocardiography [5]. As shown in Fig 1, echocardiography-derived eccentricity index (EI) was defined as the ratio of D2/D1, where D1 is the LV short-axis diameter perpendicular to the septum and D2 is the LV short-axis diameter parallel to the septum [6]. We measured the EI at its maximum during end-systolic phase. Furthermore, 4D-echocardiography of the RV was performed to calculate right-ventricular volumes and ejection fraction. Short axis views were acquired at base, papillary muscle and apical levels in tissue velocity imaging (TVI) mode at the smallest angle feasible to achieve maximum resolution. At least three ECG-gated heart cycles were recorded for analysis. All participants were in sinus rhythm at the time of the examination. Echocardiography was performed with a Vivid E9 using a standard M5S probe for 2D ultrasound and Doppler measurements, and a 4V probe for 4D sequences (GE Vingmed Ultrasound, Horten, Norway).

### Data analysis

All echocardiographic data were analyzed on an EchoPAC workstation (BT12, GE Vingmed Ultrasound, Horten, Norway). For RV measurement, the TomTec-Arena^TM^ software package (version 1.1, TomTec Imaging Systems GmbH, Unterschleissheim, Germany) was used according to vendor instructions. For calculation of left ventricular rotation, 2D speckle tracking (STE) was performed at all three acquisition levels and the results were exported as raw data. Three consecutive equally spaced beats (less than 10% beat-to-beat variation of cycle length) were analyzed. The standard parameters of echocardiography were collected using a custom python script to construct a relational database for further analysis.

### Statistical analysis

Data were analyzed with R statistical software package (version 3.1 for Windows, R Development Core Team, Vienna, Austria) and a p<0.05 was considered statistically significant. Scalar data were examined for normality by Kolmogorov-Smirnov-test. Data from the control subjects have a narrow distribution of EI around mean. The data have three features: a) small sample size; b) inhomogeneity of variance; and c) asymmetry of distribution. We thus opted for the Kruskal-Wallis-H-Test for scalar variables. For the more complex analysis of continuous data, which is the focus of this study, we opted for a method using permutation testing. We therefore limited confounding variables and avoid further subgroups in this dataset due to the small sample size. All results are displayed as median and range.

For time dependent analysis of torsion, functional data analysis (FDA) was applied. Therefore, instantaneous net torsion angle and torsion rate were extracted and replaced by a function modeling of the data. As measurements are distributed equidistant in time, a Fourier expansion model with n = 9 basis functions was used to create a function of expected torsion and torsion rate, respectively. The cycle length T to calculate frequencies of the Fourier functions was set to 100%. Due to this functional approach, no normalization of raw data was necessary. Smooth coefficients of the expansion were estimated by minimizing the unweighted least square criterion SMSSE. This produced a single deformation pattern to each time point within the heart cycle of each patient for further analysis. To analyze the impact of left ventricular eccentricity on time dependent rotational movement, a functional linear model was fit depending on EI treated as a continuous variable. The null hypothesis was tested that rotational movements are the same regardless of EI values. Due to the functional nature of the method, theoretical distributions could not be calculated. Therefore, a permutation F-test was applied to calculate global and pointwise F-statistics. Methodological details have been published previously [7].

The study complies with specifications from the declaration of Helsinki and was approved by the local ethics committee (Ethikkommission der Ärztekammer des Saarlandes, Ref. 153/11). Written informed consent was obtained from patients and healthy volunteers.

## Results

The anthropomorphic parameters are shown in Table 1. Body weight and body surface area were similar between patients group and healthy control group. Patients and control group showed a statistically significant difference in heart rate. We also found a correlation of heart rate with both maximal torsion rate (r = 0.52, p<0.01) and maximal twist rate (r = 0.52, p<0.01). Neither torsion nor twist showed a statistically significant correlation with heart rate in this data set.

**Table 1:** Basic characteristics of PH patients and control group.

		Control n = 13		PH n = 52		
		median	range	median	range	p value
Age	[years]	41	42	55	65	0.001
Height	[cm]	178.0	30.0	164.0	34.0	<0.001
Weight	[kg]	74.0	37.0	77.5	102.0	n.s.
BSA	[sqm]	1.99	0.54	1.81	0.98	n.s.
HR	[bpm]	62	59	74	51	<0.01

BSA: body surface area. HR: heart rate. Non-parametric test was performed between the control group and the PH group.

Echocardiographic examination results are shown in Table 2. Compared to the healthy control group, septal to inferior-posterior diameter was reduced, and eccentricity index was increased in the patients’ group. This is accompanied by a diminished LV inner diameter in PH patients. Left ventricular volumes were reduced by 72 ml / 48% (EDV) and 26 ml / 54% (ESV) respectively; ejection fraction remained within normal range in PH patients (63%). Left ventricular stroke volume was reduced by 45ml (46%) in PH patients. Right ventricular volumes (RV EDV: 100 ml, and RV ESV: 72 ml) as determined by 4D echocardiography were both numerically increased in PH patients, but did not differ statistically from healthy control subjects. Right atrial area and volume were similar in PH patients and healthy controls.

**Table 2:** Cardiac dimensions and morphology.

		Control n = 13		PH n = 52		
		median	range	median	range	p value
Diameter 1	[mm]	49.16	20.43	50.99	37.13	n.s.
Diameter 2	[mm]	45.79	16.94	38.50	34.87	<0.001
EI		1.08	0.31	1.39	1.36	<0.001
LV length	[mm]	79.32	35.59	69.05	39.74	<0.001
IVSd	[mm]	11	8	11	9	n.s.
LVPWd	[mm]	10	8	10	8	n.s.
LVIDd	[mm]	51.0	18.0	42.5	29.0	<0.001
LV EDV	[ml]	138.5	120.0	72.0	127.0	<0.001
LV ESV	[ml]	57.0	47.0	26.0	34.0	<0.001
LV EF	[%]	58.0	13.0	63.0	18.0	n.s.
LV SV	[ml]	83.5	73.0	45.0	73.0	<0.001
RV EDV	[ml]	95.0	69.0	100.0	218.0	n.s.
RV ESV	[ml]	50.0	60.0	72.0	158.0	n.s.
RV EF	[%]	45.0	26.0	29.0	50.0	<0.001
RAAd	[sqcm]	19.9	8.9	23.7	34.7	n.s.
RA EDV	[ml]	59.0	45.0	85.0	183.0	n.s.

Diameter 1: the left ventricle short-axis end-diastole diameter perpendicular to the septum, Diameter 2: the left ventricle short-axis end-diastole diameter parallel to the septum, EI: eccentricity index, IVSd: interventricular septum in diastole, LVPWd: left ventricular posterior wall in diastole, LVIDd: left ventricular diameter in diastole, LV: left ventricle, RV: right ventricle, EDV: end-diastolic volume, ESV: end-systolic volume, EF: ejection fraction, SV: stroke volume, RAAd: right atrial area in diastole (planimetry), RA EDV: right atrial end-diastolic volume

As shown in Table 3, right ventricular systolic pressure (RVSP) and pulmonary artery end-diastolic pressure (PAEDP) were significantly increased in the PH group (RVSP: 64.0 vs. 29.0 mmHg, p<0.001 and PAEDP 21.6 vs. 12.2 mmHg, p<0.001). Left ventricular index of myocardial performance (LIMP) was similar between the two groups, while right ventricular index of myocardial performance (RIMP, i.e. Tei index) was markedly increased in patients with PH compared to healthy control group (0.55 vs. 0.28, p = 0.007). Conversely, tricuspid annular plane systolic excursion (TAPSE) was reduced in PH patients compared to healthy control subjects (19.0 vs. 26.5 mm, p<0.001).

**Table 3:** Parameters of cardiac function, performance, and estimated pressure gradients.

		Control (n = 13)		PH (n = 52)		
		median	range	median	range	P value
RVSP	[mmHg]	29.02	18.54	64.03	111.96	<0.001
CVP	[mmHg]	10	5	10	15	n.s.
PAEDP	[mmHg]	12.16	8.63	21.64	69.05	<0.001
RVOT V_max_	[m/sec]	0.75	1.06	0.78	0.79	n.s.
PV V_max_	[m/sec]	0.96	0.93	0.95	0.99	n.s.
TR V_max_	[m/sec]	2.18	1.15	3.66	3.56	<0.001
PR V_max_	[m/sec]	1.52	0.76	2.58	3.41	<0.001
PRend V_max_	[m/sec]	0.81	0.76	1.69	3.15	<0.001
LIMP		0.45	0.37	0.48	0.92	n.s.
RIMP		0.28	0.61	0.55	1.19	0.007
TAPSE	[mm]	26.50	9.00	19.00	22.00	<0.001

RVSP: right ventricular systolic pressure, CVP: central venous pressure by V. cava variability, PAEDP: estimated end-diastolic pulmonary artery pressure, RVOT: right ventricular outflow tract, PV: pulmonary valve, TR: tricuspid regurgitation, PR: pulmonary valve regurgitation, PRend: pulmonary valve end-diastolic regurgitation, Vmax: maximal velocity by Doppler measurement, LIMP: left ventricular index of myocardial performance, RIMP: right ventricular index of myocardial performance, TAPSE: tricuspid annular plane systolic excursion.

In patients with PH, the maximum torsion was significantly lower compared to healthy control subjects (6.86° vs. 12.00°, p = 0.027). Furthermore, the peak occurred significantly later during the heart cycle (441.0‰ vs. 339.5‰, p = 0.034). Maximum torsion and twist rate were similar between the two groups (Table 4).

**Table 4:** Parameters of twist and torsion using speckle tracking echocardiography.

		Control (n = 13)		PH (n = 52)		
		median	range	median	range	p value
TorsionMax	[degrees]	12.00	17.55	6.86	25.05	0.027
TorsionMaxPos	[‰ cycle]	339.5	240	441	762	0.034
TorsionRateMax	[degrees/sec]	81.34	111.58	55.53	134.92	n.s.
TorsionRateMaxPos	[‰ cycle]	193	155	213	942	n.s.
TwistMax	[degrees/mm]	0.15	0.27	0.13	0.34	n.s.
TwistRateMax	[degrees/sec/mm]	0.95	2.47	1.16	3.61	n.s.

Torsion signifies the net torsion angle between base and apical rotation. Twist refers to the net torsion angle standardized by LV length. The positions of the maxima are expressed as 1/1000 of the heart cycle.

**Fig 2:**
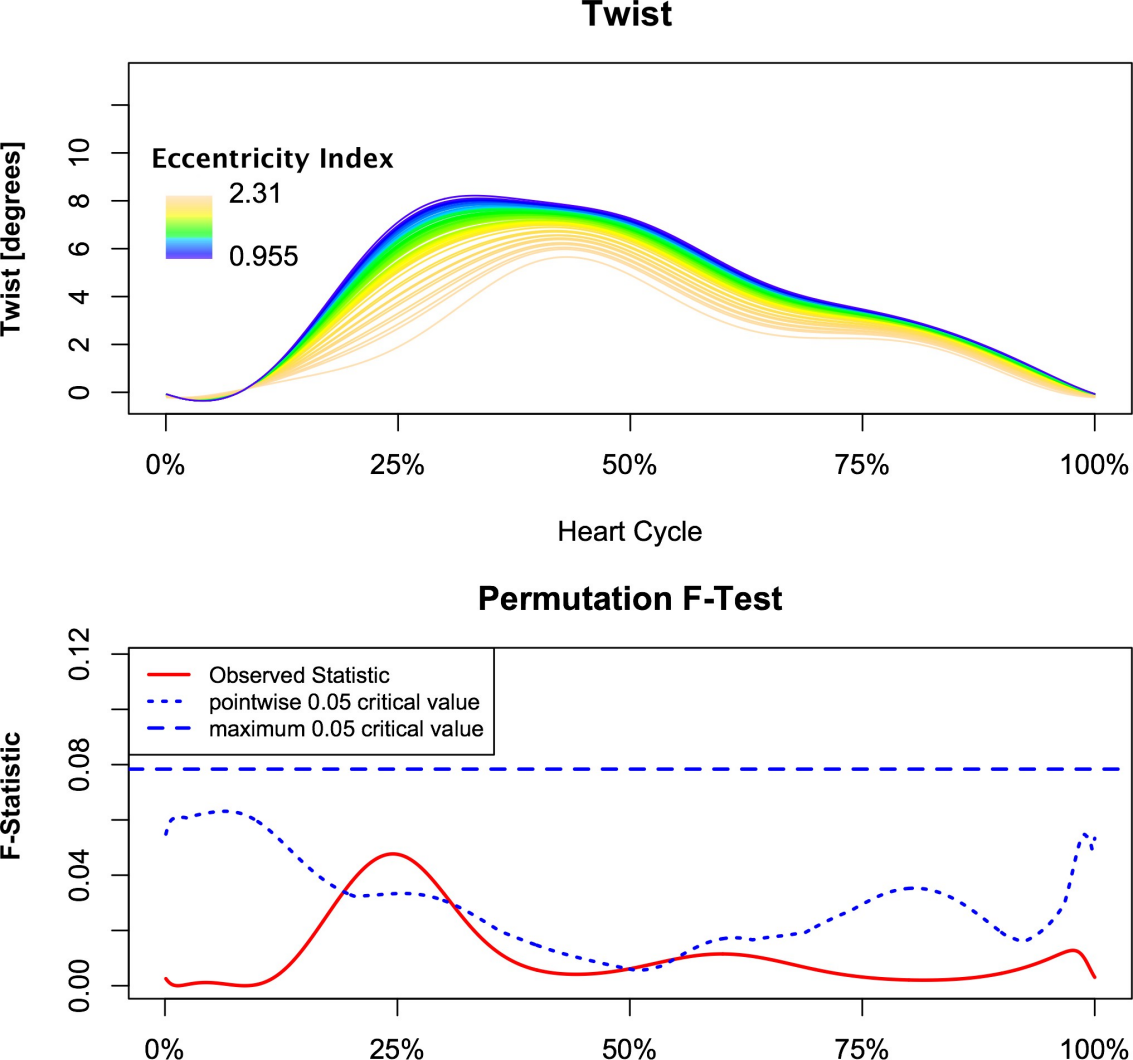
Correlation of instantaneous left ventricular torsion with left ventricular eccentricity. Patients and control subjects are included. The pointwise statistical significance is shown in the lower panel. The major effects are during initiation of the systole and peak systolic torsion. Furthermore, a delay of peak systolic torsion can be seen.

**Fig 3:**
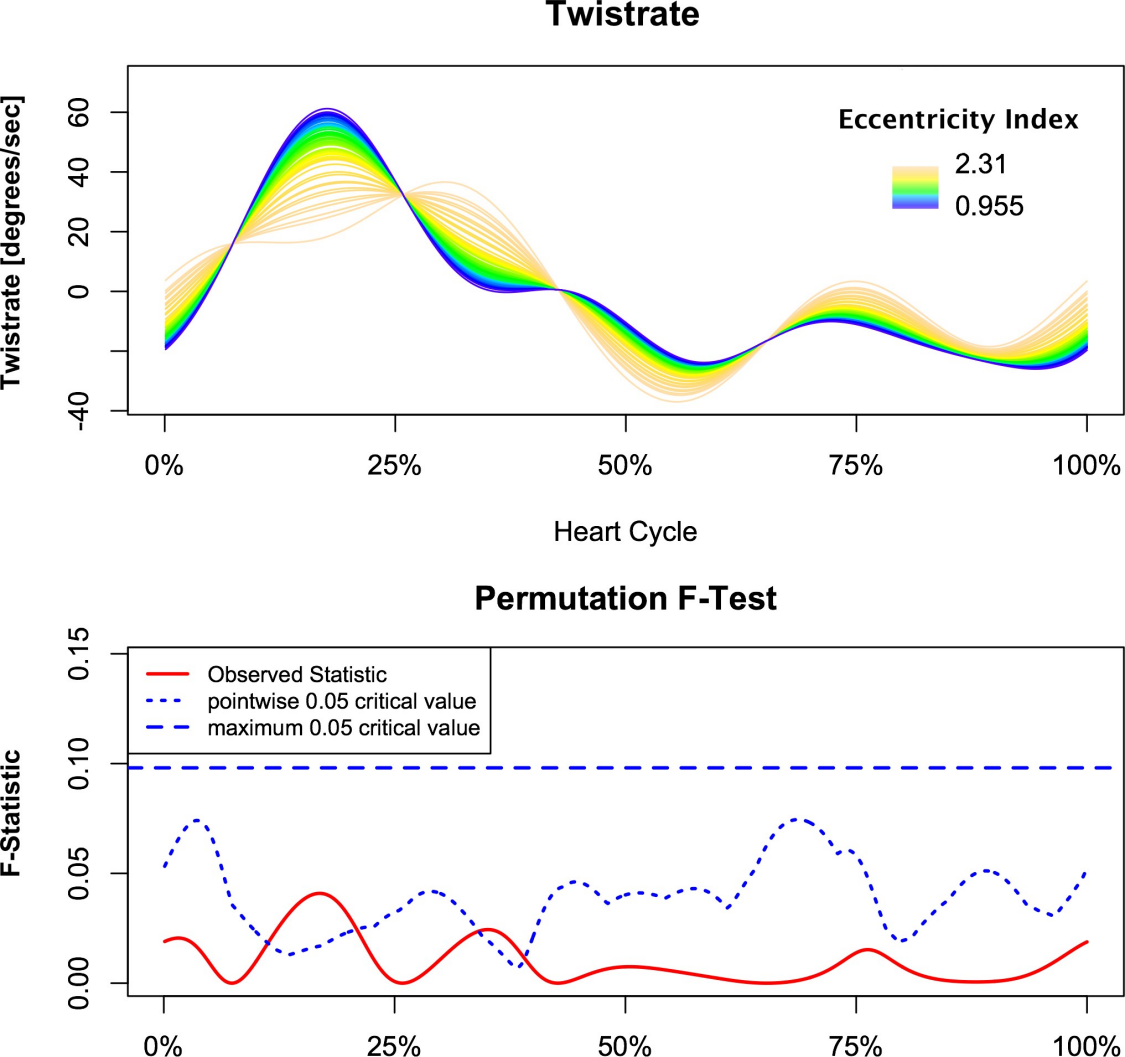
Correlation of instantaneous left ventricular torsion rate with left ventricular eccentricity. Patients and control subjects are included. The pointwise statistical significance is shown in the lower panel. The main effect is in early systole, where ventricles with low eccentricity index reach higher torsion rate and therefore have earlier and higher peak systolic torsion compares to eccentric ventricles.

**Fig 4:**
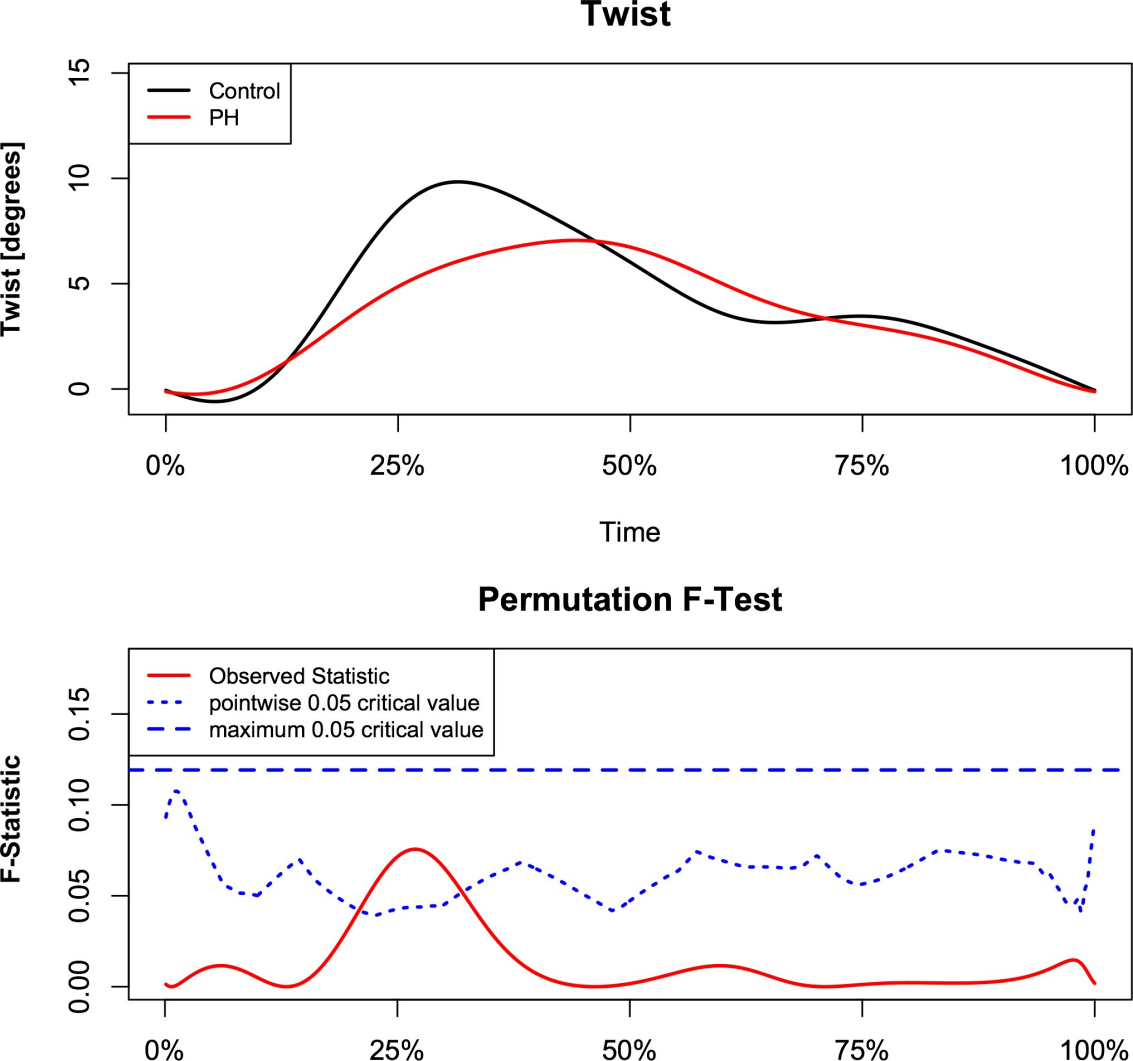
Comparison of instantaneous left ventricular torsion between PH patients and healthy control subjects. Pointwise statistical significance is shown in the lower panel.

**Fig 5:**
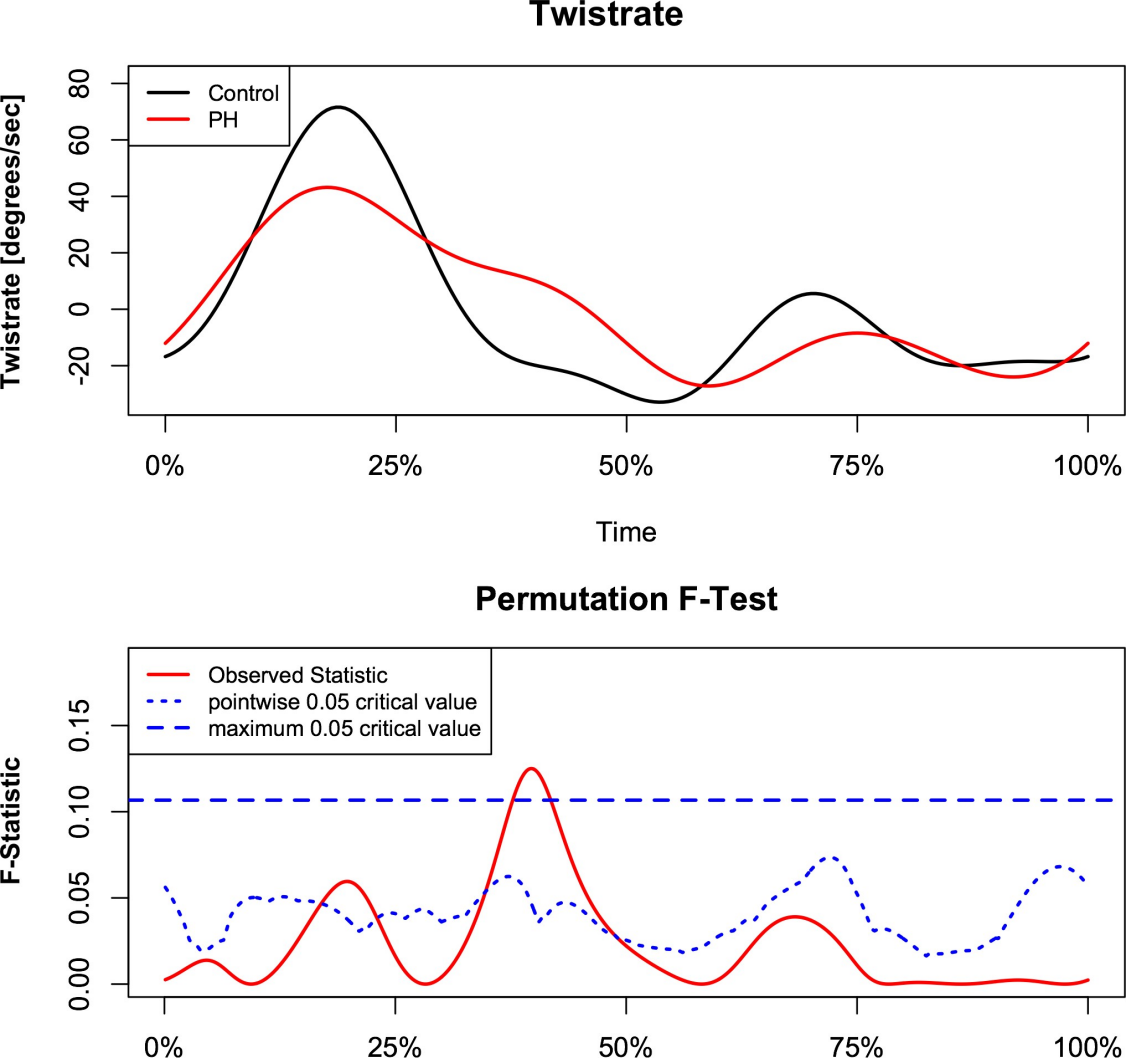
Comparison of instantaneous torsion rate between PH patients and healthy control subjects. Pointwise statistical significance is shown in the lower panel.

Analysis demonstrated a relationship between torsion and left ventricular eccentricity as resembled by EI. Figs 2 and 3 demonstrate the relationship of torsion and torsion rate over the observed range of EI with respect to time, normalized for the duration of the heart cycle in the whole study population. The F-statistics of LV torsion reaches the local significance level of 0.05 at roughly 25% of the heart cycle. Instead, F-statistics for LV torsion rate demonstrate two time intervals of significance during systole at approximately 17% and 38% of the heart cycle. This corresponds to systolic torsion and reflects the concomitant shift to the right with slightly decreased maximal torsion rate. While untwisting rate tended to be faster in early diastole in proportion to increasing EI (Fig 3). When PH patients and control subject were compared, similar intergroup differences were found in continuous torsion and torsion rate analysis. PH patients had diminished and delayed peak torsion and showed a reduced positive torsion rate during initiation and slower torsion rate at the beginning of diastole (Figs 4 and 5).

**Fig 6:**
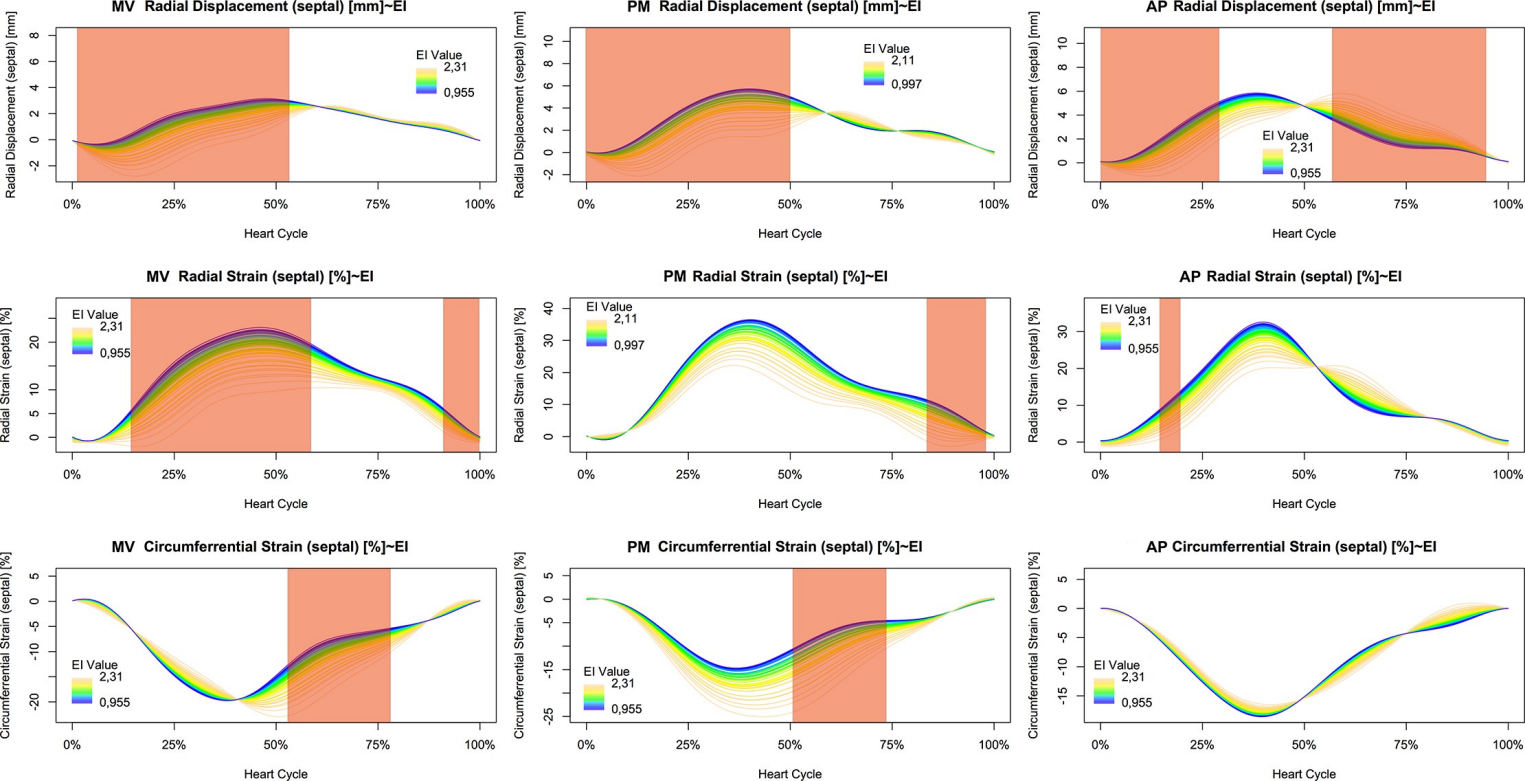
Dependence of septal movement and deformation on left ventricular eccentricity during the heart cycle. Radial displacement, circumferential strain, and radial strain are displayed at basal, mid and apical level. Statistical significance is indicated by shaded areas.

Septal movement was analyzed using displacement at mitral valve, papillary muscle, and apical short axis views. The systolic inward movement was most prominent in the mitral valve and papillary muscle levels. In contrast, differences during diastole were only detected in the apical short axis views (Fig 6, first row). Septal radial strain was markedly diminished with increasing LV eccentricity, but significance was reached only at mitral valve level and late diastole at papillary muscle level (Fig 6, mid row). Instead, septal circumferential strain at mitral valve level showed a pronounced delay of peak strain, but no marked reduction of global strain. Therefore, significance was reached during late systole and diastole. At papillary muscle level, circumferential septal strain increased with left ventricular eccentricity but showed less delay compared to the mitral valve level. Again, statistical significance was reached in late systole and early diastole. Finally, septal circumferential strain at apical level was unaltered by left ventricular eccentricity (Fig 6, last row).

Univariate analysis demonstrated negative correlations of both maximal torsion and maximal systolic torsion rate with left ventricular septal to inferior-posterior diameter (r = -0.33, p = 0.0087; r = -0.38, p = 0.002).

Stroke volume correlated negatively with eccentricity of the LV (r = -0.434, p = 0.034). This was revealed as consequence of diminished end-systolic and end-diastolic volumes as seen in the negative correlations with EI (r = -0.504, p = 0.012 and r = -0.491, p = 0.015, respectively). No correlation was found with left ventricular ejection fraction. With decreasing right ventricular ejection fraction, eccentricity of the left ventricle increased (r = -0.500, p = 0.006), while right ventricular or atrial end-systolic and end-diastolic volumes were not correlated with EI. Instead, the highest correlations of EI were seen with end-diastolic pulmonary artery pressure (r = 0.714, p<0.001) and right ventricular systolic pressure (r = 0.643, p<0.001).

Eccentricity of the left ventricle was not associated with altered diastolic function of the left ventricle by univariate analysis. However, an association of left ventricle eccentricity with right ventricular filling was seen. The tricuspid A-wave was correlated with EI (r = 0.424, p = 0.005), while tricuspid E-wave and E/A-ratio were not correlated. In time-resolved plots of left ventricular torsion by A-wave-velocity, significantly higher systolic and diastolic torsion can be seen. Untwisting of the left ventricle was faster (highly negative torsion rate) with increasing A-wave-velocity and E/A-ratio.

## Discussion

In this study, we demonstrated that alterations of the right ventricle due to PH affect left ventricular mechanics and specifically the left ventricular torsion and torsion rate. To the best of our knowledge, the only structured examination of these left ventricular characteristics until then has been provided by Puwanant et al. [8]. Regarding left ventricular torsion, an association between EI and maximal systolic torsion was described. The EI itself was found linked with systolic pulmonary artery pressure in this study. While these findings match the results presented in our own examinations, the study is limited by the nature of the data, examining singular points during the cardiac cycle. In contrast, we used techniques from the rather young field of functional statistics to analyze the rotational deformation of the left ventricle over time and to determine the exact phases during the heart cycle, at which significant changes can be expected. This methodology revealed significantly reduced net torsion of the left ventricle during systole depending on the extent of deformation as signified by the eccentricity index. In addition, a shift of the peak torsion to a later phase of the heart cycle could be demonstrated. This leads to corresponding changes in net torsion rate in the ascending and descending limp of the torsion function over time. Similar associations of left ventricular torsion with systolic function have been described in context with left heart disease and left ventricular sphericity.

In this examination, net torsion was used for time resolved analysis opposed to normalized values as described in previous studies [9]. Normalization of torsion is performed regarding diastolic left ventricular length and diameter. As ventricular length and diameter change during heart cycle and one independent variable of the analysis was the eccentricity index, which is based on the ratio of diameters of the left ventricle, normalization was not feasible. Furthermore, for the anterior and lateral walls both higher rotation and higher twist angles have been described. Earlier studies attributed this affect to misplacement of the centroid [10]. As deformation of the LV occurs in PH, the centroid can shift with increasing eccentricity index. This effect is influencing parameters normalized for both ventricle length and diameter, as those are directly related to twist angle and fiber orientation. Due to lacking mathematical models and experimental data in deformed ventricles, this was not addressed in this study.

The analysis of septal deformation demonstrated the inhomogeneity of septal mechanics due to right ventricular pressure overload and consecutive left ventricular geometric changes. While septal displacement analysis reflects the visual impression in short axis views with main changes during systole in all parts of the septum, radial and circumferential strain showed a more complex picture. Radial strain showed no delay of peak deformation and main changes were found during systole at mitral valve level. In contrast, circumferential strain in the same area was markedly delayed and pronounced. This might support the initial hypothesis of passive fiber insufficiency due to the geometric changes of the left ventricle.

Complex anatomical changes demand 4D-echocardiography for acquisition of ventricular volumes and ejection fraction. In the current investigations, we found EI to be negatively correlated with LV stroke volume. It has been published previously that in PH left ventricular volumes are diminished and associated with both reduced filling and reduced stroke volume [11]. This is in line with the observation that chronic reduced filling of the left ventricle causes hypotrophy of the left heart resulting in reduced stroke volume while maintaining ejection fraction [12]. The hypotension observed in late stage pulmonary hypertension can therefore in part be explained as a result of decreased cardiac output on top of vasoactive specific medication. The concomitant decrease of afterload might affect left ventricular rotation and therefore torsion. ESV and EDV have been used before as surrogate parameters for left ventricular loading condition [13]. In this context, ventricular volumes are not necessarily closely correlated to pressure and should therefore be used with care when drawing further conclusions. With respect to torsion, diminished left ventricular diameter and length as seen in the current study alter normalized values in the same numerical manner as twist. Additionally, the geometric alteration might disturb the diagonally oriented fibers of the inner and outer layer of the LV wall. This might result in increased net torsion due to more horizontally oriented contraction. Therefore, while increasing EI influences systolic was shown to effect net torsion negatively in the current study, other parameters associated with pulmonary hypertension might be affected differently.

The analysis of the right ventricle using 4D-echocardiography showed markedly increased right ventricular volumes and reduced ejection fraction in PH patients. Though, correlation with EI was weak. This contrasts previous experiments, where acute pulmonary hypertension was shown to lead to right sided dilation of the heart and septal shifting, which was resolved by pericardial fenestration [14]. Instead, a close correlation of EI with end-diastolic pulmonary artery pressure and right ventricular systolic pressure was found. Therefore, the hypothesis of confined space within the pericardium leading to the shift of the interventricular septum cannot be supported by the current data. On contrary, increased afterload of the right ventricle and increased preload of a failing right ventricle, appear highly associated with septal shift. This leads to the assumption, that at least end-systolic EI as used in this study reflects intraventricular pressures of the right ventricle. The resultant hypertrophy of the right ventricle and necessarily higher preload increases its stiffness and explains the association of EI with the amount of tricuspid A-wave. While we were able to clearly demonstrate the influence of EI on systolic function, a detailed analysis of diastolic function of the right ventricle was beyond the scope of the current work. However, we acknowledged the association of diastolic right ventricular function with left ventricular torsion and eccentricity.

Of note, patients with PH tend to develop peripheral right bundle branch block which has been attributed to increasing fibrosis of the moderator band [15]. This might contribute to distorted timing and interventricular interaction. Interventricular septal bowing has been linked to interventricular delays in timing [16]. On the basis of these findings, the authors hypothesized that shifts in the timing between the ventricles cause a strain gradient over the interventricular septum, resulting in the phenomenon of bowing. The key variable under examination was circumferential strain as determined by MRI. This can be used as an explanation for movement of the interventricular septum and therefore paradoxical motion. However, both systolic and diastolic D-sign and EI resemble parameters, which are not defined by their motion, but by the geometry at specific phases during the heart cycle. As left ventricular eccentricity can be observed in all phases of the heart cycle in patients with PH, this is most likely attributed to different mechanisms such as alterations of volume or pressure. Furthermore, the aforementioned conduction abnormality is anatomically located outside the interventricular septum, making direct effects on left ventricular intraventricular timing unlikely. Nevertheless, changes in the timing of pressure peaks in the different cardiac chambers might influence the overall function and specifically the mechanics of the left ventricle, which the methods used in our current study, were not able to correct for. This might contribute to the later peak of torsion and the shortened diastolic phase as seen in the continuous time-resolved torsion analysis. As increased heart rate might shorten mainly the diastole of the cardiac cycle, this might add another factor, this study did not account for.

Functional statistics permit to evaluate where and with what level of difference between groups an event occurs along the time. In the work presented, for the first time functional statistic to evaluate left ventricular torsion and torsion rate along the different phases of the heart cycle were used. This methodology allowed for detection of differences in rotational deformation of the left ventricle in patients with pulmonary hypertension. Furthermore, it provided detailed insights into segmental time-resolved wall deformation and functional mechanics. Pulmonary hypertension is not always easy to diagnose and follow up by echocardiography. Therefore, the methodology described in here provides additional parameters that can help to follow up disease progression and response to new drugs.

Regarding the acquisition of torsion rate, STE at optimized conditions was used in the current study. Temporal resolution, however, still has both an upper and a lower limit. On one hand, the minimally detectable change in location of the myocardial reflectors sets an upper limit to the frame rate. On the other hand, the lower the frame rate, the broader the time interval needed to calculate torsion rate becomes, leading to a reduction in temporal resolution. Mathematically, this becomes less important as functional statistics were used and there is only a minor impact on the parameters of the functions. Though, this might not suffice for patient to patient comparison using this technique and does not result in feasible parameters or cut-off values to use as a diagnostic tool in daily patient care.

It is to note that EI is a time-varying measurement. The time variation of LV eccentricity is incompletely understood. It has been considered that volume overload could lead to D-sign in end diastole to mid systole phase, while pressure overload could lead to D-sign in mid systole to end systole phase; however, above statements are rather based on common sense without solid support from clinical/experimental data. It is known that there is an overlap between both volume and pressure overload induced maximum eccentricity, and the overlap spectrum is wide as well. King et al. described a progression of the D-sign from end-diastole to end-systole with increasing RV to LV pressure gradient [17]. The reason for this wide variation remains unknown. Increasing pressure of the right ventricle is associated with a later peak in the TR signal and reduced dP/dt following the maximal pressure. This might contribute to an increase in the instantaneous trans-septal pressure gradient. It is possible that increasing pressure could also lead to enhanced fibrosis of the moderator band of the right ventricle, which might facilitate the development of the atypical (peripheral) right bundle branch block often seen in these patients. This again might lead to an electrical delay of the free wall of the right ventricle and might further contribute to an interventricular asynchrony and therefore transseptal pressure gradient.

The patient sample in this study was relatively small; the obtained results thus suffer some kind of selection bias. Further studies with larger samples are warranted to confirm these findings.

## Conclusions

In the present study, advanced numeric and statistical techniques were used to investigate the influence of left ventricular eccentricity on dynamic torsion mechanics, demonstrating the effects of biventricular geometric changes due to pressure overload of the right ventricle on the torsion component of the left ventricle. Analysis of the time course of torsion or determination of the eccentricity index as single parameter might proof useful in future investigations on the interaction between left and right ventricular morphology and function.

## Supporting information

S1 Data (XLSX)

S2 Data (XLSX)

S3 Data (XLSX)

S4 Data (CSV)

S5 Data (CSV)
